# 基于多源公开数据库整合分析核心基因构建肺腺癌预后风险模型

**DOI:** 10.3779/j.issn.1009-3419.2025.102.38

**Published:** 2025-10-20

**Authors:** Chengmeng WANG, Lu ZHANG, Yu ZHANG, Yu WANG, Meng WANG

**Affiliations:** ^1^300060 天津，天津医科大学肿瘤医院肺部肿瘤科，国家恶性肿瘤临床医学研究中心，天津市恶性肿瘤临床医学研究中心，天津市肿瘤防治重点实验室，天津市肺癌诊治中心（王成蒙，张钰，王勐）; ^1^Department of Lung Cancer, Tianjin Medical University Cancer Institute & Hospital; National Clinical Research Center for Cancer; Tianjin’s Clinical Research Center for Cancer; Tianjin Key Laboratory of Cancer Prevention and Therapy; Tianjin Lung Cancer Center; ^2^天津医科大学肿瘤医院肿瘤细胞生物学实验室（王成蒙，王宇）; ^2^Laboratory of Tumor Cell Biology; Tianjin Medical University Cancer Institute & Hospital; Tianjin 300060, China; ^3^300400 天津，天津市儿童医院（天津大学儿童医院），天津市儿童出生缺陷防治重点实验室（张露）; ^3^Tianjin Children’s Hospital; Children’s Hospital, Tianjin University; Tianjin Key Laboratory of Birth Defects for Prevention and Treatment, Tianjin 300400, China

**Keywords:** 肺肿瘤, 核心基因, 聚类分析, 风险模型, 预后预测, Lung neoplasms, Core genes, Cluster analysis, Risk model, Prognosis prediction

## Abstract

**背景与目的:**

肺腺癌（lung adenocarcinoma, LUAD）靶向治疗中酪氨酸激酶抑制剂（tyrosine kinase inhibitors, TKIs）耐药问题突出，亟需筛选与耐药及预后相关的关键分子标志物以指导精准治疗。本研究旨在探究LUAD TKIs耐药的分子机制，筛选核心差异表达基因（differentially expressed genes, DEGs），明确不同基因聚类与患者生存、药物反应的关联，构建并验证LUAD预后预测的风险模型，为LUAD精准治疗与预后评估提供依据。

**方法:**

整合GSE162045、GSE114647等多个LUAD相关数据集，通过韦恩图筛选核心重叠DEGs并构建基因相关性网络。采用共识聚类法对样本进行分组，结合t-SNE降维可视化验证聚类稳定性与区分度。运用京都基因与基因组百科全书（Kyoto Encyclopedia of Genes and Genomes, KEGG）与基因集富集分析（Gene Set Enrichment Analysis, GSEA）探究DEGs功能。比较不同聚类中12种药物的半抑制浓度（50% maximal inhibitory concentration, IC_50_）值，评估药物敏感性差异。通过*LASSO*回归筛选预后相关核心基因构建风险模型，并在GSE31210队列中通过桑基图、*Kaplan-Meier*生存曲线、受试者工作特征（reciever operating characteristic, ROC）曲线验证模型效能。分析关键基因在不同聚类及风险组间的表达差异，绘制单基因表达与生存关联的*Kaplan-Meier*曲线。基于多个数据集（GSE19804、GSE19188、GSE44077、GSE30219）分析PLEK2在LUAD组织中的表达，并通过Western blot检测其在表皮生长因子受体（epidermal growth factor receptor, EGFR）-TKIs耐药细胞系中的蛋白水平。

**结果:**

筛选出12个核心DEGs（如*HMGA1*、*PLEK2*等）；当聚类数（K值）为2时样本稳定分为Cluster A和Cluster B，10个核心基因在两组中表达差异显著（*P*<0.0001），且Cluster A患者总生存期（overall survival, OS）、无病生存期（disease-free survival, DFS）、无进展生存期（progression-free survival, PFS）均显著优于Cluster B。两组在*TP53*、*KRAS*、*EGFR*等高频基因突变类型上存在明显差异，KEGG富集分析显示差异基因主要富集于“细胞周期”“神经活性配体-受体相互作用”等通路。GSEA提示Cluster B与肿瘤恶性进展相关基因集显著关联。药物敏感性分析显示两聚类对10种药物的IC_50_值存在显著差异。成功构建基于9个基因的风险模型，高风险组患者死亡比例更高、生存率更低（*P*<0.0001），模型在1、3、5年的曲线下面积（area under the area, AUC）分别为0.700、0.647、0.675，GSE31210队列验证显示模型具有良好稳定性与通用性。关键基因在风险组间表达差异显著（*P*<0.0001），其中*HMGA1*、*PLEK2*高表达提示预后不良，而*ID3*、*DAPK2*与预后无关。将临床变量与*LASSO*风险评分纳入分析，单因素*Cox*分析显示风险评分与OS显著关联（HR=0.49, *P*=3.80×10^-6^）；多因素校正后，风险评分仍为独立预后因素（HR=0.57, *P*=6.40×10^-4^），具有稳定独立预测价值。公共数据集分析及Western blot实验均证实，PLEK2在LUAD组织中表达上调，且在EGFR-TKIs耐药细胞系中表达进一步升高。

**结论:**

本研究构建的风险模型可有效预测LUAD患者的预后，其中PLEK2在LUAD中高表达且与EGFR-TKIs耐药有关，可能成为潜在的预后标志物和治疗靶点。

## Abstract (en)

**Background and objective:**

Tyrosine kinase inhibitors (TKIs) resistance poses a significant challenge in the targeted therapy of lung adenocarcinoma (LUAD), highlighting the need to identify key molecular markers associated with both drug resistance and prognosis to guide precision treatment. This study aimed to elucidate the molecular mechanisms underlying TKIs resistance in LUAD, identify core differentially expressed genes (DEGs), clarify the relationships between different gene clusters and patient survival/drug response, and construct and validate a prognostic risk model for LUAD, thereby providing a foundation for precision therapy and prognostic assessment.

**Methods:**

Multiple LUAD-related datasets, including GSE162045 and GSE114647, were integrated. Core overlapping DEGs were identified using Venn diagrams, and a gene correlation network was constructed. Consensus clustering was applied for sample grouping, combined with t-SNE dimensionality reduction to visually validate clustering stability and distinctness. Kyoto Encyclopedia of Genes and Genomes (KEGG) and Gene Set Enrichment Analysis (GSEA) were performed to explore the functional enrichment of DEGs. The 50% maximal inhibitory concentration (IC_50_) values of 12 drugs were compared across clusters to evaluate differences in drug sensitivity. Prognosis-related core genes were selected via *LASSO* regression to construct a risk model, whose performance was subsequently validated in the GSE31210 cohort using Sankey diagrams, *Kaplan-Meier* survival curves, and receiver operating characteristic (ROC) curves. Expression differences of core genes among clusters and between risk groups were analyzed, and *Kaplan-Meier* curves were plotted to assess the association between individual gene expression and survival. The expression of PLEK2 in LUAD tissues was analyzed based on multiple datasets (GSE19804, GSE19188, GSE44077, GSE30219), and its protein level in epidermal growth factor receptor (EGFR)-TKIs-resistant LUAD cell lines was detected by Western blot.

**Results:**

Twelve core DEGs (*e.g.*, *HMGA1*, *PLEK2*) were identified. When the cluster number (K) was set to 2, samples were stably divided into Cluster A and Cluster B. The expression of 10 core genes was significantly different between the two clusters (*P*<0.0001), and the patients in Cluster A exhibited significantly better overall survival (OS), disease-free survival (DFS), and progression-free survival (PFS) compared to those in Cluster B. Notable differences were observed in the mutation profiles of high-frequency genes such as *TP53*, *KRAS* and *EGFR* between the clusters. KEGG enrichment analysis revealed that the DEGs were primarily enriched in pathways such as "Cell cycle" and "Neuroactive ligand-receptor interaction". GSEA indicated significant associations with gene sets related to the malignant progression of tumors. Drug sensitivity analysis demonstrated significant differences in IC_50_ values for the 10 drugs between the two clusters. A risk model based on 9 genes was successfully constructed. Patients in the high-risk group had a higher proportion of deaths and significantly lower survival probability (*P*<0.0001). The area under the curve (AUC) values for the model at 1, 3 and 5 years were 0.700, 0.647 and 0.675, respectively. Validation in the GSE31210 cohort confirmed the model's stability and generalizability. The expression of core genes differed significantly between risk groups (*P*<0.0001). High expression of *HMGA1* and *PLEK2* was associated with poor prognosis, whereas the expression of *ID3* and *DAPK2* showed no significant association with prognosis. Univariate *Cox* regression incorporating clinical variables and the *LASSO* risk score demonstrated that the risk score was significantly associated with OS (HR=0.49, *P*=3.80×10^-6^). After multivariate adjustment, the risk score remained an independent prognostic factor (HR=0.57, *P*=6.40×10^-4^), exhibiting stable independent predictive value. Analysis of public datasets and Western blot experiments confirmed that PLEK2 expression was upregulated in LUAD tissues and further elevated in EGFR-TKIs-resistant cell lines.

**Conclusion:**

The risk model constructed in this study effectively predicts the prognosis of LUAD patients. PLEK2 is highly expressed in LUAD and associated with EGFR-TKIs resistance, suggesting its potential as a prognostic biomarker and therapeutic target.

肺癌作为全球癌症死亡的首要原因，其高度异质性导致患者预后差异显著，完善精准诊疗体系是改善临床结局的关键^[1-3]^。在肺腺癌（lung adenocarcinoma, LUAD）这一主要病理亚型中，针对表皮生长因子受体（epidermal growth factor receptor, *EGFR*）、间变性淋巴瘤激酶（anaplastic lymphoma kinase, *ALK*）等基因的酪氨酸激酶抑制剂（tyrosine kinase inhibitors, TKIs）靶向治疗虽革新诊疗模式，但临床痛点突出：据报道，*EGFR*突变LUAD患者接受第一代TKIs治疗后，10-14个月内50%-60%会出现耐药^[4,5]^，且奥希替尼等第三代TKIs治疗后仍会因*EGFR* C797S突变、间质上皮细胞转化因子（mesenchymal-epithelial transition factor, *MET*）扩增等机制出现继发耐药^[6,7]^，叠加不同患者对TKIs的治疗反应差异显著、生物标志物检测医保覆盖不足等问题，严重制约精准医疗的临床转化^[8-10]^。

分子分型是解析LUAD质性、指导TKIs精准治疗的核心基础。传统病理分型仅依赖组织形态学特征，无法全面反映肿瘤分子层面的复杂性；多组学技术虽推动分子分型研究，如Wang等^[11]^通过基于多源公共数据库的整合分析识别出LUAD亚型特异性脆弱位点，但现有成果仍存在明显局限，且与LUAD TKIs治疗场景的适配性不足。Lin等^[12]^开发的8基因LUAD预后模型，仅依赖癌症基因组图谱（The Cancer Genome Atlas, TCGA）单一队列完成内部验证，未通过基因表达综合数据库（Gene Expression Omnibus, GEO）等外部独立队列验证稳定性，难以排除样本偏倚对模型效能的影响，更无法确保对TKIs治疗人群的预测适用性。

预后评估是LUAD临床管理的重要环节。当前基于临床病理参数（如分期、分化程度）的预后模型，因未纳入分子层面信息，无法精准预测TKIs治疗患者的生存结局；Thai等^[13]^在LUAD领域顶级综述中也明确指出，当前研究多聚焦单一维度，缺乏“基因型-表型-疗效”的跨维度关联分析，难以满足临床对精准诊疗工具的需求。

鉴于此，本研究聚焦LUAD患者的TKIs治疗需求，整合TCGA-LUAD转录组、临床及体细胞突变数据（mutation annotation format, MAF），以及GEO数据库中多个LUAD TKIs相关数据集（GSE162045、GSE114647等），通过基于多源公共数据库的整合分析筛选TKIs耐药相关核心差异表达基因（differentially expressed genes, DEGs）并构建基因网络；采用共识聚类实现样本稳定分型，验证其与患者生存、*TP53*/*KRAS*/*EGFR*突变谱及TKIs敏感性的关联；借助*LASSO*回归筛选预后相关基因，构建预后风险模型，并在GSE31210外部队列中验证模型效能，明确关键基因（如*PLEK2*）在预后预测与TKIs耐药中的作用。研究通过“分子分型-预后预测-TKIs敏感性关联”的三维分析框架，针对性解决现有研究“分型与TKIs用药脱节、模型缺乏外部验证、未整合耐药相关突变数据”的局限，旨在优化LUAD分子分型体系，提升TKIs治疗人群的预后评估精度，为LUAD精准治疗与TKIs耐药机制研究提供潜在靶点与理论依据。

## 1 资料与方法

### 1.1 数据下载

本研究的分析数据主要来源于公共数据库。LUAD转录组测序数据及相应的临床信息来自TCGA。从TCGA数据库下载了LUAD样本的RNA测序原始计数（count）数据和标准化的每百万转录本中基因的转录本数量（transcripts per million, TPM）表达矩阵，用于后续的表达水平分析。同时，从同一数据库获取了TCGA-LUAD队列的MAF，用于突变谱分析。此外，为进一步探讨与TKIs治疗相关的基因特征及其生物学功能，从GEO下载了多个与LUAD TKIs治疗相关的数据集，包括GSE162045、GSE114647、GSE193258、GSE75602和GSE89127。上述数据集涵盖了LUAD患者或细胞系在不同TKIs治疗条件下的测序数据，用于筛选与TKIs反应相关的关键基因并进行后续验证分析。同时又下载了GSE31210数据集，该数据集包含LUAD样本的基因表达谱及相应的预后信息，用于独立队列的验证分析。

### 1.2 细胞系

人LUAD *EGFR*突变阳性埃克替尼敏感PC-9细胞系、人LUAD *EGFR*突变阳性埃克替尼耐药PC-9-IR细胞系、人LUAD *EGFR*突变阳性阿美替尼敏感H1975细胞系、人LUAD *EGFR*突变阳性阿美替尼耐药H1975-AR细胞系均由天津医科大学肿瘤医院肿瘤细胞生物学实验室提供。

### 1.3 共识聚类分析

为探讨与TKIs相关的12个关键基因在LUAD中的表达模式及其潜在分子分型特征，基于TCGA-LUAD队列的标准化数据进行了共识聚类分析（Consensus Clustering）。使用R语言包Consensus Cluster Plus（version 1.70.0）对样本进行聚类，通过重复抽样方法评估聚类结果的稳定性。分析过程中设定聚类次数（K）为2-6，采样比例设为0.8，重复次数为1000次，以确定最优聚类数。根据累计分布函数（cumulative distribution function, CDF）曲线及其增量变化（delta area），确定最佳聚类数。

### 1.4 突变数据分析

利用TCGA-LUAD队列的MAF，对不同分型（Cluster）间的基因突变特征进行比较分析。使用R语言包maftools（version 2.16.0）对MAF格式的突变文件进行整理与可视化，绘制各分型的突变谱（oncoplot），展示突变频率最高的基因及其在不同Cluster中的分布情况。同时，比较不同Cluster中关键驱动基因（*KRAS*、*EGFR*、*ALK*、*ROS1*）的突变频率及突变类型分布，评估其在不同分型中的差异特征，以揭示潜在的分子机制差异和靶向治疗相关基因变异特征。

### 1.5 药物敏感性分析

为评估不同分型样本对靶向药物的潜在敏感性差异，基于癌症药物敏感性基因组学数据库（Genomics of Drug Sensitivity in Cancer, GDSC2）的公开数据开展药物敏感性分析。下载并读取了GDSC2数据库中的细胞系基因表达矩阵（GDSC2_Expr，RMA标准化并对数转换）及对应的药物反应数据（GDSC2_Res）。利用R语言包oncoPredict（version 1.4）的函数构建药物反应预测模型。以GDSC2的表达谱和药物反应数据作为训练集（trainingExprData与trainingPtype），以TCGA-LUAD样本的基因表达矩阵（exp）作为测试集（testExprData），进行药物敏感性预测。

### 1.6 富集分析

为探讨不同分型相关差异基因所涉及的生物学功能与信号通路，首先利用edgeR（version 3.42.0）进行差异分析，筛选阈值设定为|log₂FoldChange|>1且调整后*P*值（*P*_adj_）<0.05。利用R包clusterProfiler（version 4.10.0）对筛选出的DEGs进行京都基因与基因组百科全书（Kyoto Encyclopedia of Genes and Genomes, KEGG）功能富集分析，以识别显著富集的信号通路。最终，以*P*_adj_<0.05作为显著标准，并通过气泡图和柱状图进行可视化展示。此外，为进一步评估基因表达在通路水平的整体活性差异，基于全基因表达数据进行了基因集富集分析（Gene Set Enrichment Analysis, GSEA）。

### 1.7 *LASSO*回归分析

为筛选与预后特征显著关联的TKIs相关基因，采用*LASSO*回归分析。使用R语言包glmnet对候选基因表达矩阵进行建模。在分析过程中，将基因表达数据标准化后作为自变量，预设预后状态作为因变量。通过10折交叉验证确定最优惩罚参数λ，并筛选出在最优λ下的非零系数基因，作为具有显著预测价值的关键基因。

### 1.8 预后分析

利用survival和survminer包进行*Kaplan-Meier*生存分析并绘制曲线，通过*Log-rank*检验比较组间差异。

### 1.9 各EGFR-TKIs相关GEO数据集的分组方式和差异分析方法

为了系统筛选与EGFR-TKIs治疗有关的核心基因，本研究首先选择了3个与EGFR-TKIs药物处理相关的GEO数据集。每个数据集均根据处理条件将样本分为对照组和耐药组，并使用limma包进行差异表达分析，筛选出|log_2_FoldChange|>1且*P*_adj_<0.05的显著DEGs。

### 1.10 筛选出12个“TKIs相关核心基因”的具体流程和阈值设置

对于TCGA-LUAD数据，由于其为RNA-seq数据，因此采用edgeR包进行差异分析，将肿瘤组织与正常组织进行比较，筛选出显著DEGs（*P*_adj_<0.05, |log2FoldChange|>1）；同时结合TCGA临床信息进行单因素*Cox*回归分析，筛选出与患者总体生存显著关联的基因（*P*<0.05）。最终，将3类基因进行交集分析，得到12个既与EGFR-TKIs响应有关，又在LUAD肿瘤组织中差异表达且与预后显著关联的“TKIs相关核心基因”，为后续功能验证和潜在靶点研究提供依据。

### 1.11 训练集和验证集的划分策略、风险评分分组阈值的确定方法

在模型构建中，本研究采用TCGA-LUAD队列作为训练集，以构建基于关键基因的风险预测模型；并选取独立的外部队列GSE31210作为验证集，用于评估模型在不同人群中的稳健性和可推广性。关于风险评分分组阈值，本研究采用中位数作为划分标准，将患者分为高风险组和低风险组。这一阈值具有简洁、稳定且适用于不同数据集的特性，是肿瘤预后模型研究中常用的分组方式，从而确保模型分组的可重复性与可靠性。

### 1.12 PC-9、PC-9-IR、H1975、H1975-AR 4个细胞系模型的具体培养制备过程

人LUAD细胞系PC-9与H1975购自美国典型培养物保藏中心（ATCC），并经短串联重复序列（short tandem repeat, STR）鉴定。PC-9与H1975细胞系经梯度浓度递增法成功构建成为PC-9-IR与H1975-AR细胞系。经检测，所用细胞无支原体污染。细胞培养于完全型1640培养基（美国康宁公司），该培养基添加10%胎牛血清（德国PAN-Seratech公司）及1%青霉素-链霉素溶液（美国海克隆公司）；培养环境为37 ^o^C、含5% CO_2_的饱和湿度培养箱。当细胞融合度达到80%时进行传代，培养基每2日更换1次。

### 1.13 Western blot检测PLEK2的蛋白表达

用蛋白裂解液和上样缓冲液处理细胞，获得样品并测定总蛋白浓度。在电泳槽中进行聚丙烯酰胺凝胶电泳，然后在冰上转印至聚偏二氟乙烯膜上，3%牛血清白蛋白封闭2 h。加入相应一抗，4 ^o^C孵育过夜。TBST溶液洗涤3次，加入二抗，孵育1 h。PLEK2抗体（#11685-1-AP, Proteintech）、β-actin（#4967S, CST）抗体浓度均为1:1000，二抗浓度均为1:4000。TBST洗涤3次，利用化学发光显影液进行曝光显影。以β-actin为参照。

### 1.14 统计学分析

本研究中所有统计分析均使用R software（version 4.4.3）完成。连续性变量以均数±标准差或中位数（四分位数）描述，组间比较采用*Student’s t*检验或*Wilcoxon*秩和检验（取决于数据分布）。所有统计检验均为双侧检验。对于多重比较分析，采用*Benjamini-Hochberg*方法对*P*值进行校正。*P*_adj_<0.05被认为具有统计学差异。

## 2 结果

### 2.1 基于TKIs治疗关键基因的分子分型揭示LUAD亚型间的预后与突变差异

通过对GSE162045、GSE114647、GSE193258、GSE75602、GSE89127等多个LUAD TKIs治疗相关数据集进行整合分析，利用韦恩图可视化呈现多组DEGs的交集情况，据此筛选出12个核心重叠差DEGs（*CENPE*、*MFAP3L*、*HMGA1*、*ID3*、*PLEK2*、*ARRB1*、*SPDL1*、*ORC1*、*POLE2*、*PLK4*、*DAPK2*、*NUPR1*），并进一步构建基因相关性网络，直观呈现了这些核心基因（如*HMGA1*、*PLEK2*、*ARRB1*、*DAPK2*等）之间的潜在调控与关联模式。采用共识聚类方法对样本进行分组，结果显示当聚类数（K=2）时，共识矩阵呈现明显的类内高一致性特征，t-SNE降维可视化分析进一步验证了聚类的有效性，样本清晰地聚为Cluster A和Cluster B两个簇。对12个核心基因在Cluster A和Cluster B中的表达水平进行分析，结果显示10种基因在两个聚类中的表达均存在显著差异（*P*<0.0001）（图1A）。*Kaplan-Meier*生存分析结果显示Cluster A和Cluster B在总生存期（overall survival, OS）、无病生存期（disease free survival, DFS）和无进展生存期（progression-free survival, PFS）方面均存在显著差异（图1B-1D），其中Cluster A患者的生存预后显著优于Cluster B患者。基因突变热图展示了LUAD患者中高频突变基因（*TP53*、*TTN*、*MUC16*等）的突变分布（图1E），桑基图显示两组在*KRAS*、*EGFR*、*ALK*等基因突变类型和频率上存在明显差异（图1F）。

**图1:**
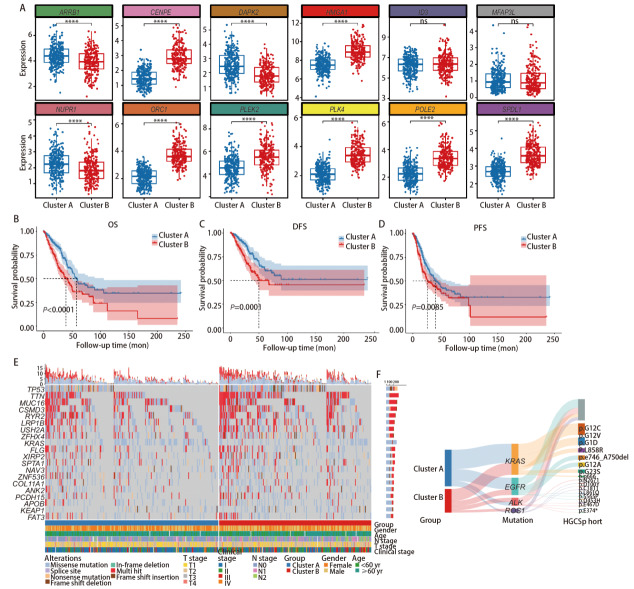
LUAD核心基因、分型与生存及突变的关联。A：箱线散点图。展示了12个候选基因（ARRB1、CENPE、DAPK2、HMGA1、ID3、MFAP3、NUPR1、ORC1、PLEK2、PLK4、POLE2和SPDL1）在Cluster A和Cluster B之间的表达水平；B-D：Kaplan-Meier生存曲线图，展示了Cluster A（蓝色）和 Cluster B（红色）OS、DFS及PFS方面的差异；E：基因突变谱与临床特征关联热图，展示EGFR-TKIs耐药LUAD患者中高频突变基因（TP53、TTN、MUC16等）的突变分布，以及突变类型（错义突变、无义突变等）与临床特征（分组、性别、年龄、TNM分期）的关联；F：桑基图，展示了Cluster A（蓝色）和 Cluster B（红色）之间的基因突变分布。

### 2.2 LUAD分子分型的功能机制与药物敏感性关联

为探究不同Cluster之间的生物差异基因，我们通过差异富集分析发现差异基因显著富集于“神经活性配体-受体相互作用”“细胞周期通路”等多条通路（图2A），这可能反映了不同Cluster之间对药物敏感性存在差异。GSEA分析显示HALLMARK_MYC_TARGETS_V1、HALLMARK_E2F_TARGETS、HALLMARK_G2M_CHECKPOINT等多个与肿瘤恶性进展有关的信号通路在Cluster B显著富集，提示Cluster B患者有着更差的预后，这与前述分析相呼应（图2B）。对12种药物的半抑制浓度（50% maximal inhibitory concentration, IC_50_）值进行比较显示Cluster A和Cluster B在多数药物的敏感性上存在显著差异（图2C），其中Cluster A对阿昔替尼、多拉米胺、帕博西尼、司美替尼、曲美替尼更敏感，提示该亚型患者可优先选择上述药物进行治疗；Cluster B对克唑替尼、厄洛替尼、吉非替尼、奥希替尼、赛沃替尼更敏感，提示该亚型患者可优先选择上述药物治疗。GSEA分析提示不同聚类在药物代谢和筛选与EGFR-TKIs敏感/耐药相关的候选基因上存在差异（图2D）。上述结果来源于体外细胞系数据的外推，只能作为潜在参考，不能直接等同于临床用药推荐。

**图2:**
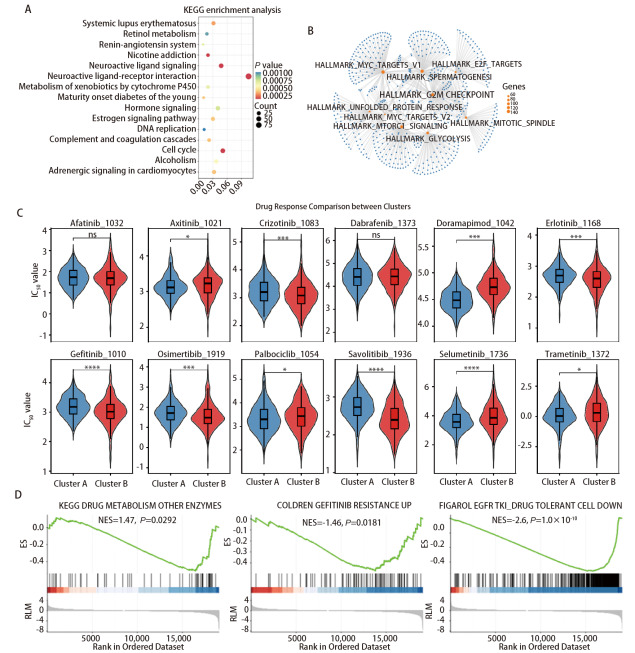
LUAD分型的通路、基因集与药物反应及耐药机制。A：KEGG通路富集分析气泡图；B：Hallmark基因集GSEA富集通路的网络图，展示显著富集通路之间的相互关联；C：小提琴箱线图比较了Cluster A（蓝色）和Cluster B（红色）对12种药物阿法替尼、阿昔替尼、克唑替尼、达拉非尼、多拉米胺、厄洛替尼、吉非替尼、奥希替尼、帕博西尼、赛沃替尼、司美替尼、曲美替尼的反应（IC_50 _值）。通过Wilcoxon秩和检验评估统计学显著性，*P<0.05，**P<0.01，***P<0.001，****P<0.0001，提示两个聚类在药物敏感性上存在显著差异；D：LUAD TKIs耐药相关基因集的GSEA富集结果图。

### 2.3 TKIs耐药相关基因预后模型构建与评估

通过*LASSO*回归分析筛选出与患者预后显著相关的核心基因。基于这些基因构建风险评分模型，并据此将患者划分为高、低风险组。风险评分分布显示两组间分界清晰，高风险组患者死亡比例显著高于低风险组。热图结果显示，关键基因（如*MFAP3L*、*NUPR1*等）在高、低风险组中呈明显差异表达，为风险分组的分子基础提供支持。*Kaplan-Meier*生存曲线（图3A）显示，高风险组患者生存率显著低于低风险组（*P*<0.0001）。受试者工作特征（reciever operating characterics, ROC）曲线（图3B）表明，模型在1、3、5年的曲线下面积（area under the curve, AUC）分别为0.700、0.647、0.675，提示模型具有较好的预后预测效能。*Kaplan-Meier*生存曲线再次验证高风险组预后更差（*P*<0.0001）（图3C）；ROC曲线显示模型在验证队列中仍具有一定预测能力，说明模型具有较好的稳定性和通用性（图3D）。对关键基因（*HMGA1*、*ID3*、*PLEK2*等）在高低风险组的表达水平进行分析（图3E），箱线小提琴图显示所有基因在高低风险组的表达均存在极显著差异（*P*<0.0001），进一步验证了这些基因为风险模型的核心标志物。针对每个关键基因，绘制其高、低表达组的*Kaplan-Meier*生存曲线（图3F）。结果显示，*HMGA1*、*ARRB1*、*PLEK2*等基因的高表达组患者生存预后显著更差（如*PLEK2*表达组*P*=0.0044），而*ID3*、*DAPK2*等基因的表达与生存预后无显著关联（如*ID3*表达组*P*=0.1600），明确了不同基因在预后中的特异性作用。将年龄、性别、T/N分期等临床变量与本研究基于*LASSO*构建的风险评分分组一同纳入分析（图3G）。单因素*Cox*结果显示，多项经典预后指标（如N分期、T分期）均与总体生存显著关联，同时，风险评分也表现出高度显著的预后价值（HR=0.49, 95%CI: 0.36-0.66, *P=*3.80×10^-6^）。在进一步的多因素分析中（图3H），将所有*P*<0.05的变量纳入同一模型，以控制潜在混杂因素。结果显示，在校正T/N分期等临床因素后，风险评分仍然是独立的预后因素（HR=0.57, 95%CI: 0.42-0.79, *P*=6.40×10^-4^）。在多因素模型中，仅IV期与T3仍具有统计学意义，而风险评分的独立预后效力依然稳定。以上结果充分表明：本研究构建的*LASSO*预后模型在校正年龄、性别及T/N分期等变量后仍具有显著的预后预测价值，因此可作为独立的预后风险因素。

**图3:**
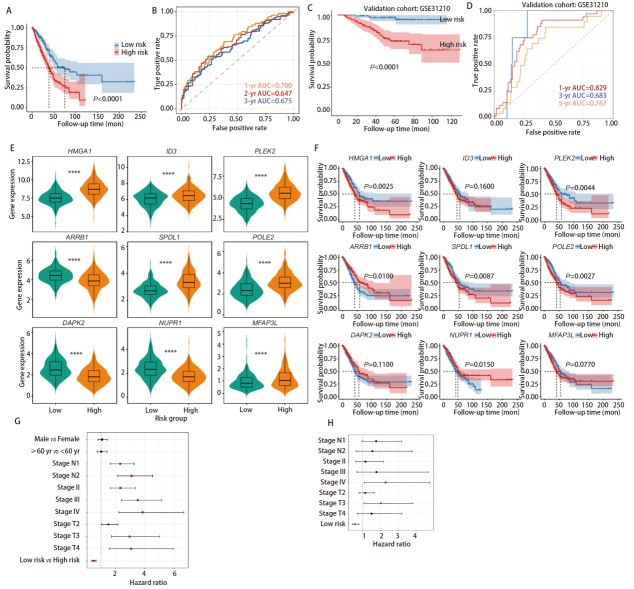
LUAD预后风险模型研究。A：Kaplan-Meier生存曲线图。展示低风险组（蓝色）和高风险组（红色）患者的生存率；B：ROC曲线，评估风险模型在1、3和5年的预测效能，对应的AUC分别为0.700、0.647、0.675；C：验证队列（GSE31210）中低风险组（蓝色）和高风险组（红色）的Kaplan-Meier生存曲线图；D：验证队列（GSE31210）的ROC曲线图，评估风险模型在1、3和5年的预测效能，对应的AUC分别为0.829、0.683、0.767；E：风险分组中关键基因的表达差异箱线图，展示9个关键基因（HMGA1、ID3、PLEK2、ARRB1、SPDL1、POLE2、DAPK2、NUPR1、MFAP3L）在低风险组（青绿色）和高风险组（橙色）中的表达水平差异；F：单个关键基因表达与生存预后的Kaplan-Meier分析图；G：单因素生存分析图；H：多因素生存分析图。

### 2.4 单基因*PLEK2*在LUAD数据库及细胞系水平的验证

通过多个LUAD数据集GSE19804、GSE19188、GSE44077、GSE30219的分析发现，PLEK2在LUAD组织中表达水平均高于癌旁组织（图4A），提示PLEK2在LUAD可能作为促癌基因参与LUAD的发生发展，且有望成为诊断标志物与治疗靶点。通过Western blot实验，发现PLEK2在埃克替尼LUAD耐药细胞系PC-9-IR中的表达水平高于在LUAD野生型细胞系PC-9中的表达水平；同时发现PLEK2在阿美替尼LUAD耐药细胞系H1975-AR中的表达水平高于在LUAD野生型细胞系H1975中的表达水平（图4）。上述结果表明，PLEK2在LUAD中高表达，且与EGFR-TKIs耐药密切关联，可能作为LUAD诊断标志物、预后预测指标及耐药逆转靶点。

**图4:**
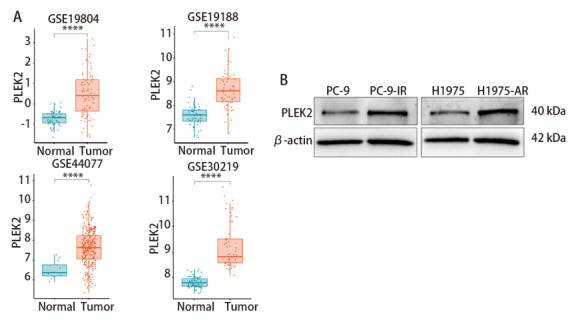
PLEK2在LUAD数据库及细胞系蛋白水平的验证。A：PLEK2在LUAD数据集GSE19804、GSE19188、GSE44077、GSE30219中的表达水平；B：PLEK2在LUAD埃克替尼敏感细胞系PC-9与LUAD埃克替尼耐药细胞系PC-9-IR中的表达水平；PLEK2在LUAD阿美替尼敏感细胞系H1975与LUAD阿美替尼耐药细胞系H1975-AR中的表达水平。

## 3 讨论

LUAD是全球发病率和死亡率最高的恶性肿瘤之一，其异质性强、治疗耐药性高，导致患者预后差异显著^[14]^。2022年中国LUAD新发病例约为106.06万例，因LUAD死亡约73.33万例，LUAD是中国癌症负担最重的病种，发病和死亡均居首位，并且其发病率仍有上升趋势^[15]^。近年来，多组学整合分析已成为挖掘肿瘤关键驱动基因、揭示分子分层及潜在治疗靶点的重要手段^[16]^。本研究基于转录组、基因组及临床数据的整合分析，成功筛选出12个与TKIs治疗效果显著相关的基因，进一步行LUAD亚型分析，在阐述了不同亚型之间的差异（突变差异、预后差异、药物敏感性差异、核心通路差异）之后，进行了关键基因的筛选，最终筛选出9个关键基因并构建风险模型，包括*ARRB1*、*DAPK2*、*HMGA1*、*ID3*、*MFAP3L*、*NUPR1*、*PLEK2*、*POLE2*和*SPDL1*。这些结果提示我们，这些核心基因并非孤立存在，可能共同构成调控LUAD进展及TKIs耐药的复杂分子网络。

对这9个关键基因进行分析发现，大体可将它们分为3类。第一类主要参与细胞增殖、凋亡与自噬调控，包括*DAPK2*与*NUPR1*：这两个基因都参与了细胞自噬过程的调控，但作用方式不同。*DAPK2*是一个保护性基因，通常参与调控细胞凋亡、自噬和代谢等过程，DAPK2下调可以导致失巢凋亡抵抗，进而导致肿瘤细胞EGFR-TKIs耐药性和肺转移能力增强^[17]^。NUPR1相反，其在非小细胞肺癌（non-small cell lung cancer, NSCLC）中高表达会抑制自噬溶酶体的正常功能，导致细胞内废物堆积和功能紊乱，从而促进肿瘤存活，NUPR1高表达患者OS更差^[18]^。另外，该类基因还包括*HMGA1*与*ID3*：它们是强大的转录调控因子，通过影响大量下游基因的表达来驱动肿瘤发展。*HMGA1*被证实是LUAD中的危险基因，它通过改变染色体结构，促进多个癌基因的转录^[19]^。*ID3*作为分化抑制因子，其高表达与LUAD的淋巴结转移和更差的分化程度显著关联，提示其在LUAD的侵袭和转移中扮演重要角色^[20]^。第二类主要参与调控肿瘤微环境及免疫应答，包括*ARRB1*与*SPDL1*：ARRB1的促癌作用与其可变剪接形式密切相关，其ARRB1-S异构体，可以显著加速肺鳞癌的进展^[21]^。SPDL1可能与肿瘤的染色体不稳定性和免疫调节有关，是LUAD的一个潜在预后标志物^[22]^。该类基因还包括*MFAP3L*与*PLEK2*：MFAP3L在LUAD中与间充质干细胞介导的肿瘤微环境重塑有关，它可能通过激活ERK信号通路来影响肿瘤发展^[23]^。PLEK2在LUAD组织中高表达，并且是患者预后不良的独立危险因素，它与LUAD的TNM分期和淋巴结转移存在显著关联，但其具体机制尚不明确^[24]^。第三类主要负责基因组完整性维持，包括*POLE2*，作为DNA复制复合物的关键组分，POLE2的正常功能对于维持基因组的稳定性至关重要，其高表达与患者的临床病理特征和不良预后密切关联^[25]^。综上所述，基于多源公共数据库的整合分析筛选出的这9个基因，从细胞内在机制（增殖、凋亡、自噬、基因组稳定）到外部微环境调控，构成了一个复杂的分子网络，共同影响LUAD的预后。

综上所述，本研究基于多源公共数据库的整合分析成功构建了基于9个核心基因的LUAD预后模型，并突出强调了PLEK2在预测患者预后及调控肿瘤恶性行为中的关键作用。这些发现为LUAD的分子分型与精准治疗提供了新的候选靶点。未来的研究将聚焦于PLEK2调控LUAD转移的具体分子通路，并评估其作为治疗靶点联合免疫检查点抑制剂或化疗药物的临床应用潜力。

值得注意的是，本研究揭示了PLEK2在LUAD预后分层中的核心地位。既往研究虽已报道PLEK2在造血系统肿瘤及部分实体瘤中的作用，但其在LUAD的具体机制尚不明确。本研究通过整合蛋白互作网络与共表达分析，推测PLEK2可能通过调控细胞骨架重组、整合素信号或与其他核心基因（如*HMGA1*、*ID3*）协同作用，影响上皮-间质转化进程，从而促进LUAD侵袭与转移。此外，NUPR1作为一种应激相关转录因子，可能在肿瘤微环境调控中与PLEK2形成反馈环路，进一步增强肿瘤细胞的适应性生存能力。
